# PLA_2_-like proteins myotoxic mechanism: a dynamic model description

**DOI:** 10.1038/s41598-017-15614-z

**Published:** 2017-11-14

**Authors:** Rafael J. Borges, Ney Lemke, Marcos R. M. Fontes

**Affiliations:** Departamento de Física e Biofísica, Instituto de Biociências, Universidade Estadual Paulista (UNESP), Botucatu, SP Brazil

## Abstract

Phospholipase A_2_-like (PLA_2_-like) proteins contribute to the development of muscle necrosis in Viperidae snake bites and are not efficiently neutralized by current antivenom treatments. The toxic mechanisms of PLA_2_-like proteins are devoid of catalytic activity and not yet fully understood, although structural and functional experiments suggest a dimeric assembly and that the C-terminal residues are essential to myotoxicity. Herein, we characterized the functional mechanism of bothropic PLA_2_-like structures related to global and local measurements using the available models in the Protein Data Bank and normal mode molecular dynamics (NM-MD). Those measurements include: (i) new geometric descriptions between their monomers, based on Euler angles; (ii) characterizations of canonical and non-canonical conformations of the C-terminal residues; (iii) accessibility of the hydrophobic channel; (iv) inspection of ligands; and (v) distance of clustered residues to toxin interface of interaction. Thus, we described the allosteric activation of PLA_2_-like proteins and hypothesized that the natural movement between monomers, calculated from NM-MD, is related to their membrane disruption mechanism, which is important for future studies of the inhibition process. These methods and strategies can be applied to other proteins to help understand their mechanisms of action.

## Introduction

Phospholipase A_2_-like (PLA_2_-like) proteins, also known as homologue-phospholipase A_2_ or K49-PLA_2_s, are widespread in the snake venom of Viperidae family members. PLA_2_-like proteins conserve the general phospholipase A_2_ scaffold, but they possess natural mutations in the calcium binding loop that preclude phospholipid hydrolysis^1^. Despite not being enzymes, PLA_2_-like proteins disrupt sarcolemma integrity by disrupting ionic homeostasis^2^. As a consequence, calcium influx induces hypercontraction of myofilaments, mitochondrial damage and activation of cytosolic enzymes, which increase cell damage and lead to muscle necrosis^2,3^. In Viperidae snake bites, muscle necrosis occurs due to PLA_2_-like proteins, phospholipases A_2_ and metalloproteinases, which is a problem with current antivenom treatment, as this injury is not easily reversed^4^. This is particularly alarming in Central and South America, where most accidents are caused by snakes from the *Bothrops* genus (American lance heads)^5^.

PLA_2_-like proteins are myotoxic, and the broad spectrum of toxicity observed in *in vitro* experiments impacts different human cells, bacteria, fungi and parasitic organisms^6^. Understanding these molecular mechanisms may lead to the development of molecules against pathogenic agents^7^. With regard to human diseases, inflammation, autoimmune disorders and cancer have been correlated with excess of endogenous phospholipases A_2_; therefore, understanding the associated activities of snake venom toxins may also support the development of strategies to suppress this hyperactivity^8^.

The disturbance of membranes by PLA_2_-like proteins has been associated with C-terminal residues^9,10^ and is enhanced if the quaternary structure is a homodimeric assembly^11,12^. Under physiological conditions, dimers are observed by dynamic light scattering^13–15^, non-reducing SDS-PAGE results^16–20^, and SAXS experiments^21^. Two different dimers have been proposed according to crystallography experiments: a large dimer with an interface composed of β-wings and a compact dimer with an interface composed of calcium binding loops. In this article, we assume that the former dimeric assembly is the biologically relevant configuration based on SAXS experiments^21^.

Based on the compact dimer and PLA_2_-like dimeric structures, dos Santos *et al*. proposed a PLA_2_-like interface binding surface (iFace)^22^. This proposition was inspired by a picture proposed by Bahnson^23^ in which sulphates ions form a plane in PLA_2_ dimeric crystal structures and mimic the phosphate head groups of membrane phospholipids. In the PLA_2_-like models, these sulphates interact with a cluster of basic residues on the C-terminus, called the membrane docking site (MDoS)^14^. The PLA_2_-like iFace is formed after these protein monomers are reoriented due to the entrance of hydrophobic molecules into the toxin hydrophobic channel^14,22,24^. With this allosteric activation, a cluster of hydrophobic residues is rearranged in both monomers to form the membrane disruption site (MDiS)^24^, which interacts with phospholipids and perturbs membrane integrity^14^.

Independent research groups characterized the different states of PLA_2_-like crystal structures by measuring the angles between their monomers. Da Silva-Giotto *et al*. and Magro *et al*. hypothesized that the flexibility observed in the different arrangements of monomers, characterized by a single angle measurement, is related to the disruption of the membrane^25,26^. Dos Santos *et al*. characterized allosteric activation by the toxin by classifying models into either inactive or active states based on a two-angle description^22^. This quaternary structural flexibility and its relationship to function can be evaluated using molecular dynamics (MD) with normal mode (NM) analysis, since low-frequency NM can describe real-world protein motions and relate them to fundamental biological properties^27,28^.

Herein, we characterize allosteric activation of PLA_2_-like proteins and the membrane disruption mechanism using global and local measurements of the available bothropic PLA_2_-like structures and corresponding MD-NM analyses. These measurements include: (i) new geometric descriptions of monomer orientation based on Euler angles; (ii) characterizations of canonical and non-canonical conformations of the C-terminal residues; (iii) accessibility of the hydrophobic channel; (iv) inspection of ligands; and (v) distance of clustered residues to the toxin interface to the membrane. By using these integrated analyses, we expand the description of the PLA_2_-like protein action mechanism, which may be important in understanding the inhibition process.

## Results and Discussion

### Tertiary structure variability: local measurement using the Cα and Cβ distances

**Figure 1:**
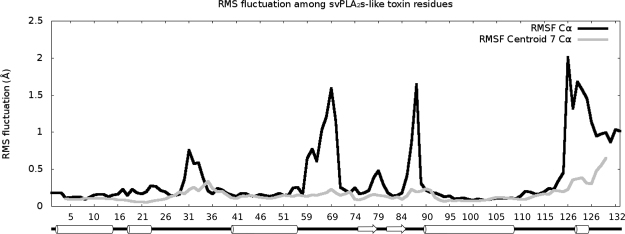
Graph of the RMSF in Å by residues of available bothropic PLA_2_-like crystallographic structures. The RMSF values of Cα and centroid of 7 consecutive Cα are coloured in black and grey, respectively. Secondary structure is shown below residue numbers with helices and sheets represented by cylinders and arrows, respectively (extracted from PDB).

PLA_2_-like proteins are small (molecular weight of approximately 14 kDa), with seven disulfide bridges and the following secondary structural elements: a N-terminal helix, putative calcium binding loop residues, two long antiparallel α-helices at the core of the protein that make up the hydrophobic channels, two antiparallel β-sheets known as β wings, one short 3_10_ helix and C-terminal loop region. Among the PLA_2_-like models, two specific regions have greater variation, with root-mean-square fluctuations (RMSF) of Cα values (calculation description is provided in Material and Methods - geometrical description between identical monomers) above 1.5 Å (black line in Fig. 1). Flexible regions are usually related to functions, as dynamic structural alterations are part of substrate binding and enzymatic actions or interactions^29^. First, the labile region at residues 86 and 87 (with 2.2 and 1.6 Å variations, respectively) was previously reported as a region that oscillates between two metastable conformations^25^. A second region, which contains the most variable residues, is in the C-terminal region of the toxin (black line in Fig. 1), where two residues (121 and 125) were suggested to be responsible for toxin-induced membrane disruption and named MDiS^14^.

MDiS is composed of residues 121 and 125, which are hydrophobic and exposed to the solvent in a short 3_10_ helix, together with residues 122 and 123^24^. This secondary structure is stabilized by one hydrogen bond between the main chain atoms of residues 121 and 125^24^. K122 is the only conserved C-terminal residue among PLA_2_-like proteins, although the hydrophobic character of residues 121 and 125 is also conserved^14^. MDiS is characterized by a Cβ distance between residues 121 and 125 (dMDiS) below 5.5 Å. In active state structures, both monomers are similar with this canonical conformation of MDiS (Tables 1 and 2). In inactive state structures, monomers are asymmetrical, with one in a non-canonical conformation and a dMDiS higher than 9 Å, as these residues are in an extended loop structure (Tables 1 and 2)^24^. Inact is the only inactive state structure with a non-canonical conformation, with a dMDiS of 8.5 Å; MDiS is in an intermediate state^24^.

**Table 1:** Summary of local and global measurements of PLA_2_-like crystallographic models.

State	PDBs	Ligands	Angles (°)			dMDiS (Å)			Tunnel (Å^3^)		toxin	Res	SG
						CanMon		N-CanMon	Monomer				
			Sp	Ts	Ap	A	B	A	A	B			
Inactive	1PA0*	—	141	26	54	—	4.8	10.8§	0	331	Bnsp-VII	2.2	P3_1_21
	2Q2J	*1 sulfate/2 TRIS*	141	27	53	—	4.9	10.9	0	267	PrTX-I	1.65	P3_1_21
	3 HZD	—	141	27	54	—	4.8	11.2	0	270^#^	BthTX-I	1.91	P3_1_21
	3I3H	—	140	27	53	—	4.6	9.3	0	251	BthTX-I	2.17	P3_1_21
	4K09	—	142	27	52	—	4.5	10.8	0	233	BbTX-II	2.11	P3_1_21
	4WTB*	3 zinc/*2 sulfates*	141	27	54	—	4.7	10.3	0	265	BthTX-I	2.16	P3_1_21
	2H8I (inact)	**1 PEG**	141	27	55	—	4.9	8.5	0	242^#^	BthTX-I	1.9	P3_1_21
Active	1QLL (acti3)	**2** ***n*** **-tridecanoic acids**	163	20	81	5.2	5.2	—	241	206	PrTX-II	2.04	P2_1_
	4KF3	**4 PEGs**/*6 isopropanol*	169	24	47	4.4	4.4	—	385	405	MjTX-II	1.92	P2_1_2_1_2_1_
	1XXS* (acti2)	**4 stearic acids**/*5 sulfate*	170	25	45	4.7	4.4	—	405	446	MjTX-II	1.8	P2_1_2_1_2_1_
	4YV5*	2 suramin/**3 PEGs**/*7 sulfates*	170	24	47	4.9	5	—	408	406	MjTX-II	1.9	P2_1_2_1_2_1_
	1Y4L*	1 suramin/**2 PEGs**/*5 isopropanol*	167	23	43	4.6	4.8	—	483	471	BaspTX-II	1.7	P2_1_2_1_2_1_
	3QNL	1 rosmarinic acid/**1 PEG**/*8 isopropanol*	173	24	34	4.8	5.2	—	459	484	BthTX-I	1.77	P2_1_2_1_2_1_
	4YZ7	1 aristolochic acid/**1 PEG**/*5 sulfates*	181	28	27	4.7	4.8	—	364	417	PrTX-I	1.95	P2_1_2_1_2
	4YU7	4 caffeic acids/**3 PEGs**/*1 sulfate*	177	27	27	4.7	5	—	509	433	PrTX-I	1.65	P2_1_
	2OK9	2 BPBs/*2 isopropanol*	180	29	25	4.5	5.3	—	458	387	PrTX-I	2.34	P2_1_
	3CYL	**2 a-tocopherols/1 PEG**/*5 sulfates*	177	28	27	4.5	5.3	—	532	406	PrTX-I	1.87	P2_1_
	3CXI	**2 a-tocopherols/1 PEG**/*4 sulfates*	178	28	26	4.7	5.1	—	415	432	BthTX-I	1.83	P2_1_
	4K06	**3 PEGs**/*5 sulfates*	178	28	27	5.1	4.9	—	458	429	BbTX-II	2.08	P2_1_
	3IQ3 (acti1)	**3 PEGs**/*2 sulfates*	177	27	27	4.7	5.2	—	473	391	BthTX-I	1.55	P2_1_
	3MLM	**2 myristic acids**/*4 sulfates*	178	28	28	4.6	5.4	—	312	460	BnIV	2.2	P2_1_

The abbreviations are related to roll angle (R), twist angle (Ts), tilt angle (Ti), the distance between the two Cβ MDiS residues (dMDiS), canonical monomer (CanMon), non-canonical monomer (N-CanMon), resolution (Res), Space Group (SG), polyethylene glycol (PEG), and *p*-bromophenacyl bromide (BPB). The toxins (TX) Bnsp, Pr, Bth, Bb, Mj, Basp, and Bn are purified from the venom of *Bothrops pauloensis*, *Bothrops pirajai*, *Bothrops jararacussu*, *Bothrops brazili*, *Bothrops moojeni*, *Bothrops asper* and *Bothrops neuwiedi*, respectively. In Ligands column, PLA2-like protein inhibitors, negative and hydrophobic natural compounds are in dashed, italic, and bold font, respectively. *Chain letter A and B were inverted. ^#^Side chain of H120 had to be remodeled for tunnel calculation. ^§^125 Cβ was absent in model, and it was manually generated.

**Table 2:** Summary of local and global measurements of PLA_2_-like crystallographic models.

State	Ligands	PDBs	Angles			dMDiS (Å)			Tunnel (Å^3^)	
						CanMon		N-CanMon	Monomer	
			Roll	Twist	Tilt	A	B	A	A	B
INACTIVE	—	1PA0, 2Q2J, 3HZD, 3I3H, 4K09, 4WTB, 2H8I	**141** (0.6)	**27** (0.4)	**54** (1.0)	—	**4.7** (0.2)	**10.3** (0.9)	**0**	**266** (29.6)
ACTIVE	Hydrophobic and negative molecules	All below	**174** (5.5)	**26** (2.6)	**37** (15.4)	**4.7** (0.2)	**5.0** (0.3)	—	**422** (78.5)	**412** (66.2)
		3QNL, 4YZ7, 4YU7, 2OK9, 3CYL, 3CXI, 4K06, 3IQ3, 3MLM	**178** (2.2)	**27** (1.4)	**28** (2.6)	**4.7** (0.2)	**5.1** (0.2)	—	**442** (69.0)	**427** (31.3)
		4KF3, 1XXS, 4YV5, 1Y4L	**169** (1.4)	**24** (0.8)	**46** (1.9)	**4.7** (0.2)	**4.7** (0.3)	—	**420** (43.1)	**432** (32.3)
		1QLL	**163**	**20**	**81**	**5.2**	**5.2**	—	**241**	**206**

The abbreviations are related to the distance between the two Cβ MDiS residues (dMDiS), canonical monomer (CanMon), and non-canonical monomer (N-CanMon). Inactive state represents the structures whose PDB IDs are: 1PA0*, 2Q2J, 3HZD, 3I3H, 4K09, 4WTB*, 2H8I. The active state represents the structures whose PDB IDs are: 4KF3, 4YV5*, 1XXS*, 1Y4L*, 3QNL, 4YZ7, 3CYL, 3CXI, 4YU7, 2OK9, 4K06, 3IQ3, 3MLM. *Chain letter A and B were inverted.

### Characterization and accessibility of the hydrophobic channel of PLA_2_-like toxins

Bothropic PLA_2_-like proteins have a cavity that is surrounded by positive and hydrophobic residues that participate in the binding of fatty acids^1^. The entrance of fatty acids into the hydrophobic channel of the toxin is hypothesized to be essential to induce dimer reorientation from the inactive to active state, a key step of the myotoxic mechanism of action^14,22^. To better characterize the allosteric activation induced by the entrance of hydrophobic molecules, we evaluated the accessibility of the hydrophobic channel through the iFace of the protein using *CAVER software*.

All canonical monomers contain a hydrophobic channel that is accessible through C-terminal residues. The PLA_2_-like structures in the inactive state only contain the accessible hydrophobic channel in monomers that are in the canonical conformation with a volume ~266 Å^3^ (Table 2). Curiously, the hydrophobic channels of the canonical monomers of BthTX-I/PEG400 and *apo1* BthTX-I are closed by the side chain of H120, whereas they are open in the available models of other bothropic PLA_2_-like proteins^24^.

PLA_2_-like structures in the active state have symmetrical monomers, and the calculated tunnel is occupied by ligands, such as fatty acids, PEG molecules or inhibitors (Supplementary Table S1). Most of the calculated hydrophobic channels have a volume of ~400 Å^3^, with the exception of PrTX-II/*n*-tridecanoic acid, whose volumes are ~223 Å^3^ (Table 2). MjTX-II has an insertion at residue 120 compared to the other bothropic toxins (BaspTX-II, BbTX-II, BnIV, BnSP-VII, BthTX-I, PrTX-I and PrTX-II); thus, instead of the hydrophobic channel exiting through the middle region of the N-terminal helix, it is shifted to the beginning of the N-terminal helix^15^. Despite these differences, all of the calculated hydrophobic channels structurally connect the C-terminal region of one monomer to the N-terminal region and interior of the two parallel α-helices of the other monomer. The N-terminal region with its hydrophobic residues may participate in toxin activity^30,31^ and may be related to membrane interactions, similar to the MDiS.

### Global measurements: orientation and translations between almost identical objects

The orientation between the monomers of available PLA_2_-like models are remarkably different and are related to the symmetrical and asymmetrical hydrophobic channel accessibility of active and inactive states structures, respectively. Previous efforts attempted to characterize the different orientations of models by one^25^ or two angles descriptions^22^. Herein, we update this angle description with a more intuitive, general and informative methodology. Since these structures are homodimers, we characterize the monomers orientation by describing the necessary rotation in Euler angles to superpose one monomer onto the other.

**Figure 2:**
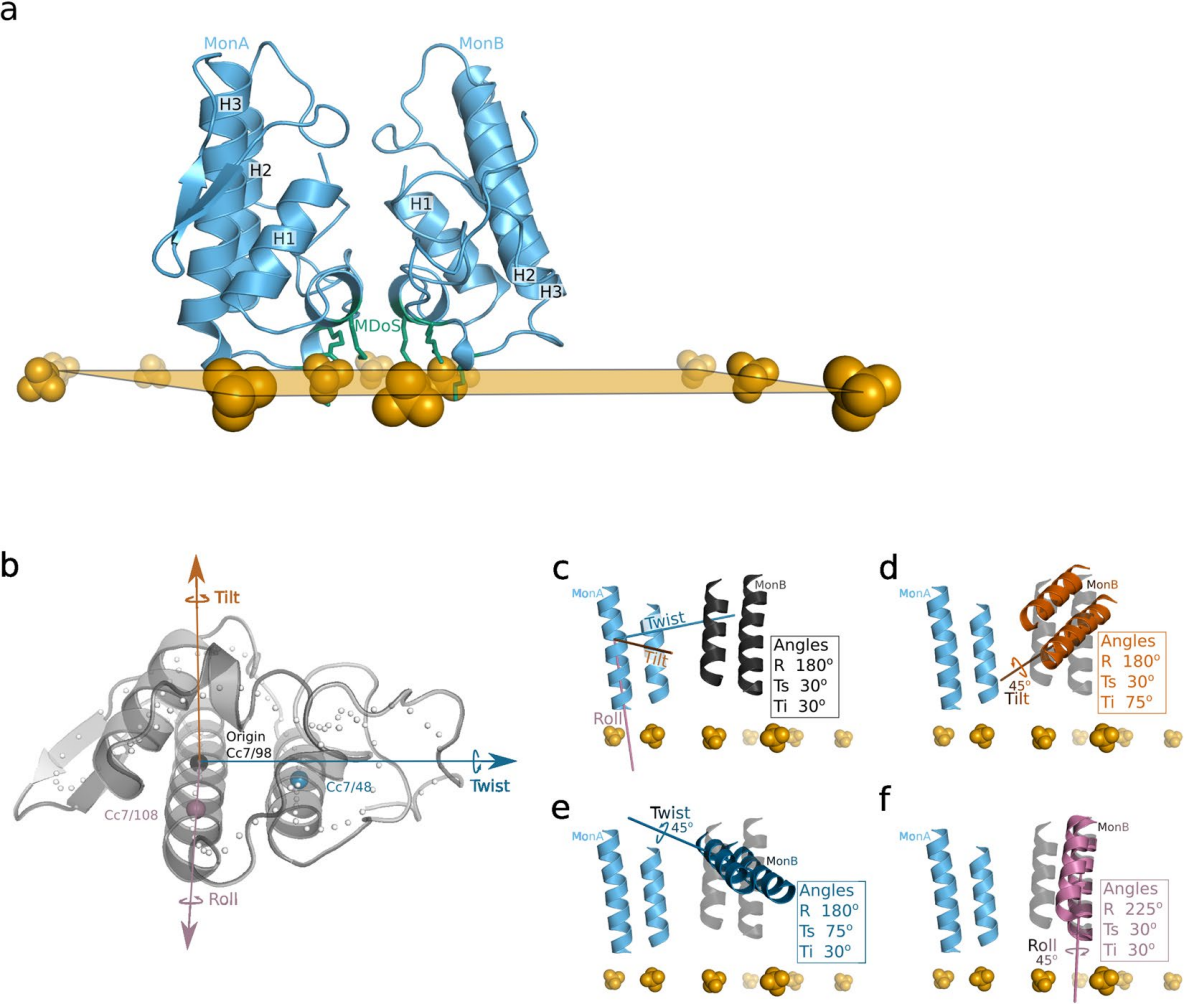
Representation of the PLA_2_-like protein iFace and proposed axes of movement. Sulphate ions are represented as orange spheres and compose a plane that indicates the PLA_2_-like iFace (in **a**). N- and C-terminal residues compose this iFace, which includes a membrane docking site represented by green sticks in (**a**) that interacts with sulphates. In (**b)** the PLA_2_-like monomer is represented by a grey cartoon with Cc7 represented as spheres. The axes are established by Cc7/98 as the origin and coloured black; by Cc7/108 as the X-axis and coloured magenta; by a perpendicular vector to the X-axis, in the plane composed by X-axis and Cc7/48 sphere, as the Y-axis and coloured dark blue; and the normal vector to the XY plane coloured vermilion. In (**c**–**f**) the two antiparallel helices are illustrated to indicate different orientations of monomer A (light blue) corresponding to monomer B; the calculated angles are shown in the box. Tilt is coloured in vermilion, twist in dark blue and roll in dark blue. In (**c**–**f**) monomer B, coloured in black, is close to the orientation of BnIV/myristic acid structure (PDB id: 3MLM), which is established as a reference to show a difference of 45° in each of the axes of movement. The axes in (**d**–**f**) are shown as illustrations of the rotation that describes monomer B in transparent black relative to the other monomer, which is not transparent.

Han *et al*. used a similar approach to study the interdomain geometry between two domains of homologous proteins^32^. They described domain motion with a single translation and single rotation. Hayward wrote the *DYNDOM* software (fizz.cmp.uea.ac.uk/dyndom) to determine the hinge axes and measure closure or twist motions in a single angle description by using two conformations of the same protein^33^. With more than 20 available crystallographic structures, our approach differs, as we distinguish PLA_2_-like models using replicable and independent metrics based on Euler angles (Fig. 2).

In our angle description and inspired by the DNA angular base pair parameters^34^, we called the angles tilt, twist and roll for the X, Y and Z axes, respectively (Fig. 2b–f and the calculation description is provided in Material and Methods - geometrical description between identical monomers). The X-axis is the centre of the longest α-helix (H3), the Y-axis is the vector that connects the X-axis to the other antiparallel α-helix (H2), and the Z-axis (orange axis in Fig. 2b) is the normal vector of the plane XY. Among the evaluated dimers, twist (Ts) is the angle with the lowest divergence and a value close to 25° in all samples (Tables 1 and 2). The roll angles (R) separate inactive and active states. The former is composed of the *apo* BnSP-VII, *apo* PrTX-I, *apo1*&2 BthTX-I, *apo* BbTX-II, BthTX-I/Zn^2+^ and BthTX-I/PEG400 (inact) structures, which have a roll of ~140° and tilt (Ti) of ~55° (Tables 1 and 2). All of these models are *apo* structures (Supplementary Table S1), with exception of BthTX-I/Zn^2+^ and *apo* PrTX-I, which have charged molecules or ions bound. Moreover, inact has a small PEG molecule inside the hydrophobic channel that induces dMDiS shortening (8.5 Å)^24^.

The structures in the active state have a roll angle greater than 160° (Tables 1 and 2). They are complexed with inhibitor molecule(s) or natural compound(s) in the hydrophobic channel (Supplementary Table S1), which agrees with the accessibility calculation of the previous sub item. Most of these structures have abundant negative molecules surrounding the protein, being the R34 and PLA_2_-like iFace [considered the side chains of residues 16, 17, 20, 115 and 118 (MDoS)] the most common region. This environment, which is rich in hydrophobic and negative molecules, may mimic a membrane interaction^23^. Roll angles of the active state close to 180° are closer to a symmetrical orientation, which may be the optimal exposure of same residues of both monomers in the protein iFace.

Most angles of active state models are similar, but they can be further separated according to their tilt angles in: 1) ~30: BthTX-I/PEG4k (acti1), BnIV/myristic acid, MTX-II/PEG4k, PrTX-I/BPB, PrTX-I/caffeic acid+PEG4k, BthTX-I/α-tocopherol, PrTX-I/α-tocopherol, PrTX-I/aristolochic acid+PEG4k, and PrTX-I/rosmarinic acid+PEG330; 2) ~50: BaspTX-II/suramin, MjTX-II/stearic acid (acti2), MjTX-II/suramin+PEG4k, and MjTX-II/PEG4k; ii.3) ~80: PrTX-II/*n*-tridecanoic acid (acti3) (Table 2). Especially for acti3, the higher tilt angle causes a shortening of the hydrophobic channels as their volumes are half of the hydrophobic channels of the other active models.

### Evaluation of the flexibility of PLA_2_-like protein in active and inactive states

Analyzing the local and global measurements of available bothropic PLA_2_-like proteins, we identified two different states based on their roll angles: i) an inactive state (roll ~ 140°) that has an asymmetrical conformation with one non-canonical monomer and one hydrophobic channel accessible and ii) an active state (roll > 160°) with symmetrical canonical monomers and with both hydrophobic channels filled by hydrophobic molecules. It was hypothesized that for these toxins, the first state is converted to the second by an allosteric activation induced by the entrance of a hydrophobic molecule into the accessible hydrophobic channel^24^.

To address the allosteric activation hypothesis *in silico*, we evaluated the flexibility via NM-MD analysis of the BthTX-I models, as both inactive and active states were structurally determined. We chose inact and acti1 as representatives of the former and latter groups, respectively. In both cases, we evaluated flexibility in absence and presence of fatty acids, whereas PEG molecules available in the model were replaced by myristic acid.

#### PLA_2_-like activation

**Figure 3:**
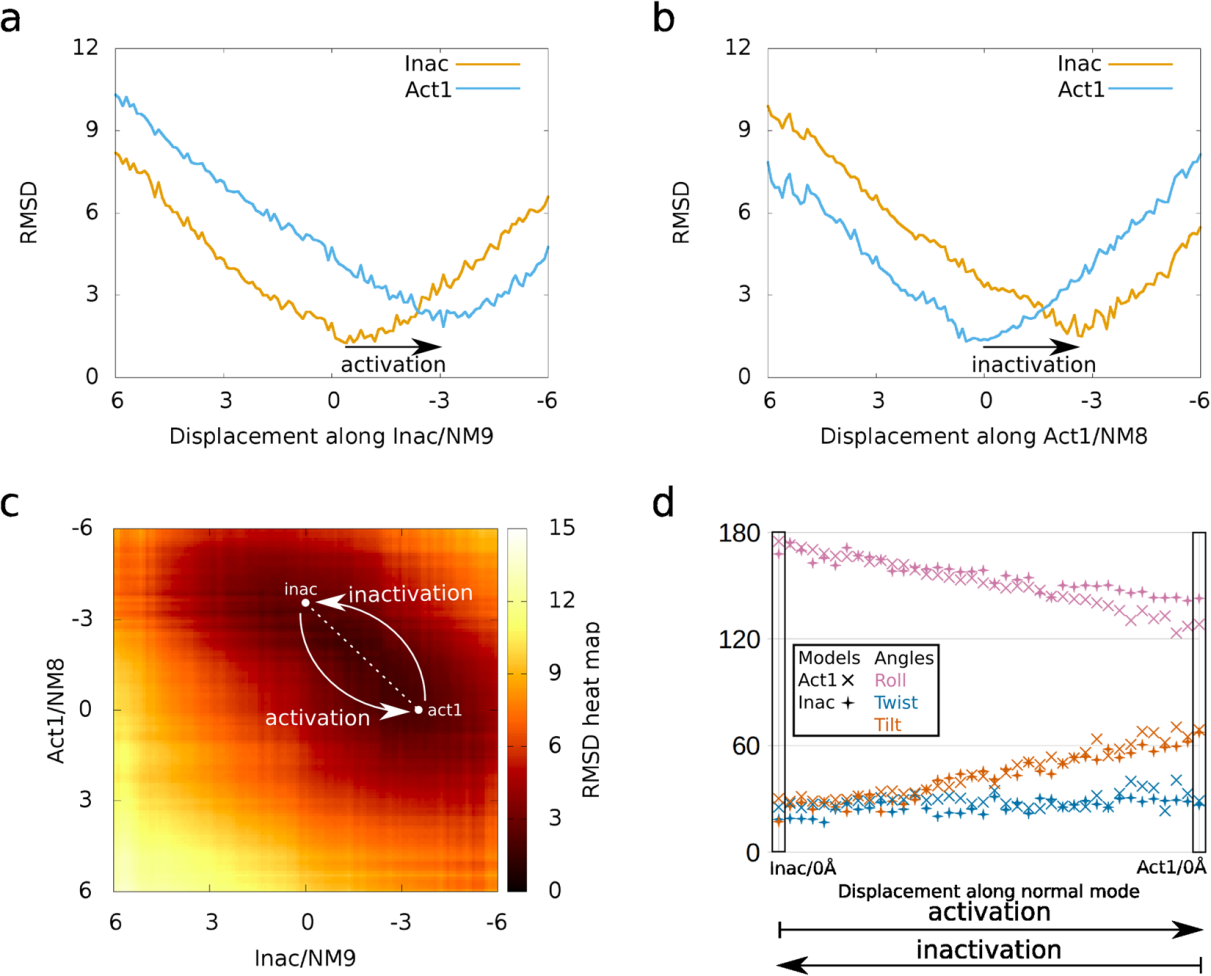
Activation and inactivation descriptions using RMSD and proposed angles. In (**a)** inact/NM9/0 Å and -3Å structures resemble inact (orange line) and acti1 (blue line), respectively, describing the BthTX-I activation process. In (**b**) acti1/NM8/−3Å and 0 Å resemble inact (orange line) and acti1 (blue line), respectively, describing the BthTX-I inactivation process. In (**c**) the RMSD heat map demonstrates darker and lighter colours corresponding to high and low similarities, respectively. The structures from the dashed white transparent line from the white circle, which starts in act/NM8/−3.7 Å and inact/NM9/0 Å and ends in acti1/NM8/0 Å and inact/NM9/−3.7 Å, are similar and describe complementarity between activation and inactivation. In (**d**) the pairs of structures delimitated by the dashed white transparent line in (**c**) have similar angles, and activation is characterized by increase of roll, stability of twist and decrease of tilt angles. In (**d**) roll, twist, and tilt are coloured magenta, dark blue and vermilion, respectively, and models of acti1 and inact are represented by X and ◆, respectively.

All of the models from inact and acti1 calculated from a ± 6.0 Å displacement of NM 7–9 had satisfactory energies, as the measured potential energies were smaller than 0.5 kcal/mol (Supplementary Fig. S2) and within order of magnitude of evaluated structures of other studies^35,36^. To observe the transition from one state to another, we compared the inact and acti1 models by superposing them on the corresponding NM-MD structures (Supplementary Table S3). Inact-FA/NM9/-3.1 Å (inact structure complexed to fatty acid minimized and displaced -3.1 Å among NM9) is the most similar to acti1, with a RMSD of 1.9 Å, and as a reference, the model itself prior to minimization has a minimum RMSD of 1.3 Å (Fig. 3a). The other calculated NMs of inact-FA have minimum RMSDs above 3 Å compared to acti1 (Supplementary Table S3). For the structures from NM calculations of *apo-*inact (without fatty acid), the most similar to acti1 had a RMSD of 2.8 Å and, as a reference, the model itself prior to minimization has a minimum RMSD of 1.2 Å (Supplementary Table S3). To confirm that lower amplitude NMs of *apo*-inact would not reach a better representation of acti1 model, the NM10–21 were also calculated, but with no better similarity than 2.8 Å. Therefore, the fatty acid in the canonical monomer of BthTX-I was fundamental for the toxin reach its active state (described by comparison with acti1) since the lowest RMSD obtained from inact-FA was almost 1 Å higher than *apo*-inact. Moreover, inact-FA/NM9 from −0.4 to −3.1 Å are the calculated structures that better describes the BthTX-I activation; such conformation changes will be further described as activation (Fig. 3a, Supplementary Fig. S4 and Supplementary Video S5).

For acti1-FA, the minimized structure on NM8/−2.7 Å is the most similar to inact, with a RMSD of 1.5 Å, and as a reference, the model itself prior to minimization has a minimum RMSD of 1.3 Å (Fig. 3b). The other calculated NMs of acti1-FA have a minimum RMSD, compared to inact, above 2.3 Å (Supplementary Table S3). For *apo-*acti1, the structure NM8/−2.1 Å is the most similar to inact with a RMSD of 1.7 Å, and as a reference, the model itself prior to minimization had a minimum RMSD of 1.8 Å (Supplementary Table S3). The presence of a fatty acid in both of canonical monomers of BthTX-I seems not essential for toxin acquiring its inactive state, as both *apo*-acti1 and acti1-FA NM8 reached similar representations of inact. Therefore, acti1-FA/NM8 from −2.7 to 0 Å are the calculated structures that better describes BthTX-I inactivation; such conformation changes will be further described as inactivation (Fig. 3b and Supplementary Video S6).

To observe whether the activation and inactivation processes were complementary, we compared them by superposing inact-FA/NM9/±6 Å and acti1-FA/NM8/±6 Å and then plotting the RMSD in a heat map (Fig. 3c), with darker colours indicating similar structures. These two processes demonstrate that there is better agreement between inact-FA/NM9/−3.7 Å and acti1-FA/NM8/0 Å to inact-FA/NM9/0 Å and acti1-FA/NM8/−3.7 Å that are 38 consecutive superpositions with an average RMSD of 2.0 Å (shown by the dashed white transparent line in Fig. 3c). The angles from these paired structures correspond, as shown in Fig. 3d, which demonstrates the activation process as a movement that increases the roll angle and decreases the tilt angle. Therefore, our results suggest that the inactive state of PLA_2_-like proteins may transition to the active state through physically plausible movements in the presence of a fatty acid in the hydrophobic channel.

#### PLA_2_-like flexibility among active state models

PLA_2_-like structures may change to an active state from an inactive state after fatty acid interaction, which is described by an increase in the roll angles. Active state models may be further separated by tilt angles, with at least one model with both hydrophobic channels occupied by a fatty acid: acti1 (BthTX-I), with ~30°; acti2 (MjTX-II), with ~50°; and acti3 (PrTX-II), with ~80° (Table 2). Are these angles exclusive to a single toxin or are they a consequence of crystal packing? To address this question *in silico*, we evaluated their flexibility with NM-MD. Only the fatty acids in the toxin hydrophobic channels were maintained in the model.

**Figure 4:**
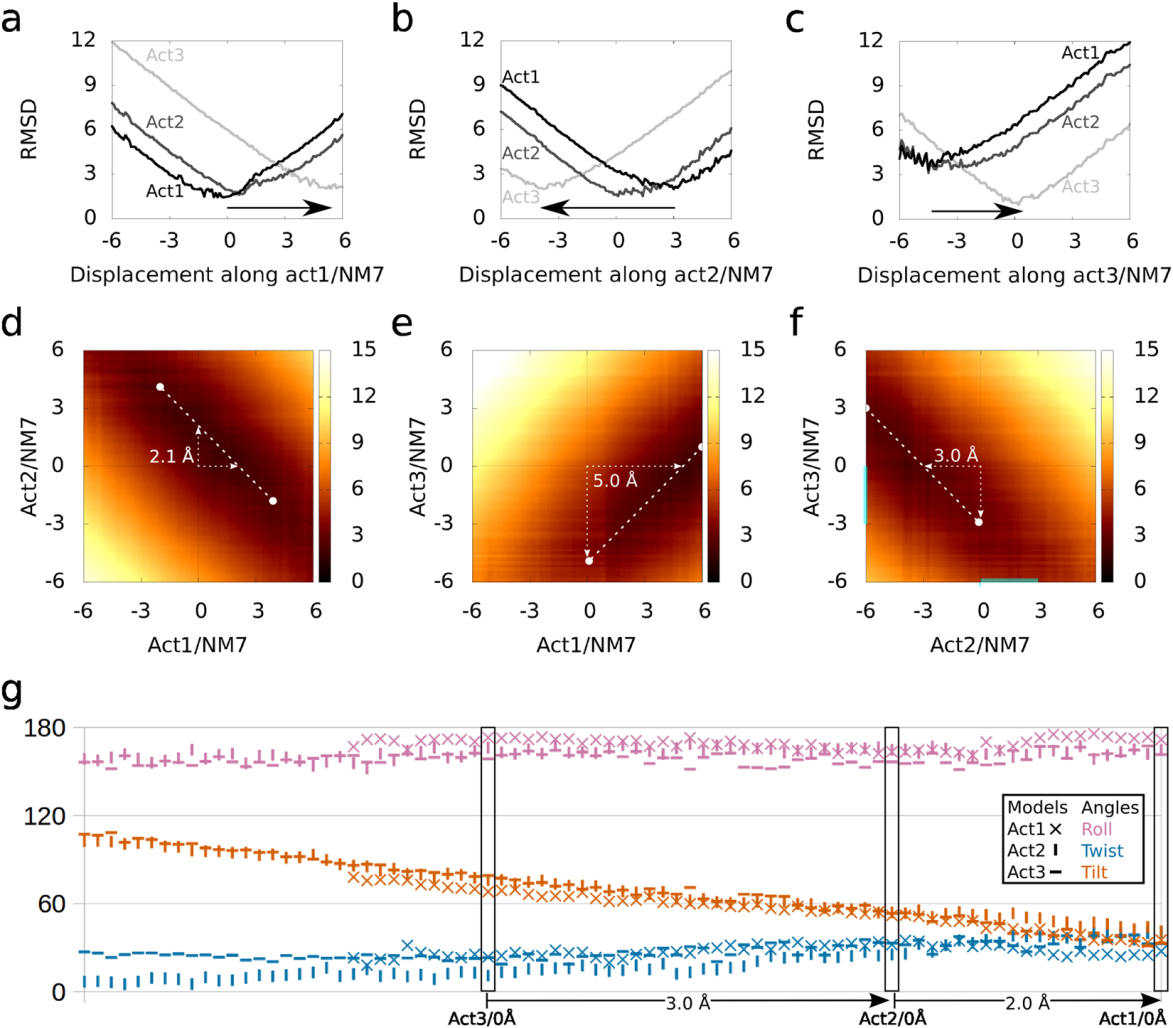
Correspondence between NM7 of acti1–3 shown with the RMSD and proposed angles. In **A**, acti1/NM7/0 Å, 0.6 Å, and 5.4 Å structures resemble acti1, acti2, and acti3 crystallographic models, respectively. In (**b)** acti2/NM7/−3.8 Å, 0 Å, and 3.0 Å structures resemble acti1, acti2, and acti3 models, respectively. In (**c)** acti3/NM7/−4.5 Å, −4.5 Å, and 0 Å structures resemble acti1, acti2, and acti3 models, respectively. In (**a**–**c**) acti3, acti2, and acti1 are coloured in light grey, dark grey, and black, respectively. In (**c**–**e**) the RMSD heat map demonstrates darker and lighter colours as high and low similarities, respectively. The dashed white lines with circles on their edges correspond to the 60 lowest consecutive superpositions; therefore, 6 Å of highly correlated pair of structures. In (**d**) the NM7s of acti1 and acti2 are similar, with a shift of 2.1 Å, as shown by dashed white arrow. In (**e**) the NM7s of acti1 and acti3 are similar, with a shift of 5.0 Å, as shown by dashed white arrow. In (**f**) the NM7s of acti2 and acti3 are similar, with a shift of −3.0 Å, as shown by the dashed white arrow. In (**g**) the angles of the acti1–3/NM7 structures are plotted following the shift of (**d**–**f**). In (**g**) roll, twist, and tilt are coloured magenta, dark blue and vermilion, respectively, and the acti1, acti2, and acti3 structures are represented by X, │, and ─, respectively.

**Figure 5:**
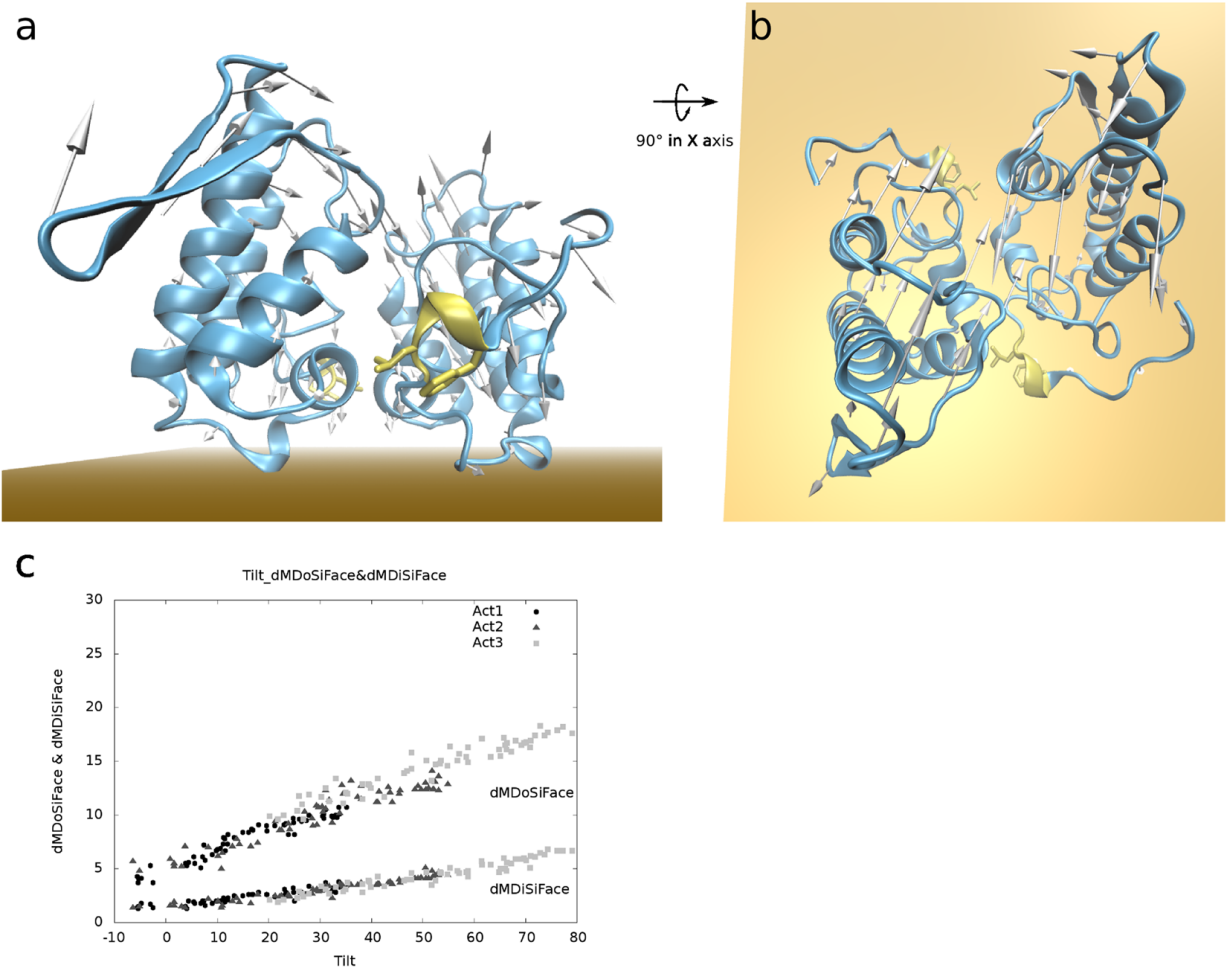
Motion describing transition between different active state structures and interacting to the membrane. In (**a**,**b**) acti1 is represented by a blue cartoon with MDiS in yellow sticks, −6 Å displacement along normal mode 7 is represented by arrows, and the membrane, by the orange plane. (**a**,**b**) Show the same representation with a 90° rotation in the horizontal axis. In (**c**) scatter plot of distances of MDoS (dMDoSiFace) and MDiS to toxin iFace (dMDiSiFace) against tilt angles. In (**c**) structures from NM7/0 to −6 Å of acti1, 2, and 3 are represented by ⦁, ▴ and ▪, respectively.

All of the models from acti1, acti2, and acti3 containing fatty acids and calculated from a ± 6.0 Å displacement of NM 7–9 had satisfactory energies, as they had potential energies of less than 0.5 kcal/mol (Supplementary Fig. S2) and within order of magnitude of evaluated structures of other studies^35,36^. Among the calculated NM, NM7 of acti1–3 were the movements that best described the conversion from one conformation to the other, as observed by RMSD comparisons (Fig. 4a–c). For acti1/NM7, the minimum RMSD for the acti2 and acti3 models were 1.6 and 2.0 Å, respectively, which corresponded to the structures acti1/NM7/0.6 Å and acti1/NM7/5.4 Å, respectively; as a reference, the model itself prior to minimization had a minimum RMSD of 1.4 Å (Supplementary Table S3). For acti2/NM7, the minimum RMSD for acti1 and acti3 were 2.0 and 2.0 Å, respectively, which corresponded to the structures acti2/NM7/3.0 Å and acti2/NM7/−3.8 Å, respectively; as a reference, the model itself prior to minimization had a minimum RMSD of 1.5 Å. For acti3/NM7, the minimum RMSD for acti1 and acti2 were 3.3 Å and 3.1 Å, respectively, which both corresponded to the structure acti3/NM7/−4.5 Å; as a reference, the model itself prior to minimization had a minimum RMSD of 1.0 Å. The other structures from calculated NMs 8 and 9 of acti1, acti2, and acti3 had minimum RMSD values compared to the independent models higher than the obtained by NM7 (Supplementary Table S3). Therefore, NM7 better describes the movements of conversion between different active state models (Fig. 5a,b and Supplementary Video S7).

The NM7 of these structures were correlated, as observed by the white dashed lines with a circle on the edges in the RMSD heat maps in Fig. 4d–f. These white dashed lines are the lowest 60 consecutive superpositions: 6 Å of highly correlated pairs of structures. The average RMSD for the superposition of these white dashed lines was 2.2, 2.6, and 2.8 Å for acti1/acti2, acti1/acti3, and acti2/acti3, respectively. Each NM7 had a different shift with respect to the other NM7, which was 2.1, 5.0, and 3.0 Å for acti1/acti2 (Fig. 4d), acti1/acti3 (Fig. 4e), and acti2/acti3 (Fig. 4f), respectively. The orientations between these paired structures, which were obtained by applying these shifts, corresponded (Fig. 4g and Supplementary Video S7). Therefore, our results suggest that the active state of PLA_2_-like structures with different tilt angles may be interchangeable by the described NM7s, which suggests that their mechanism of action is probably similar.

#### PLA_2_-like membrane disruption description

As the X-axis (measured by roll) is perpendicular to the plane of the protein iFace, the movements described by the other two orthogonal rotations, such as tilt, will change the orientations of the residues in the iFace. Therefore, the experimental flexibility observed in the tilt of the active state models and calculated NM7 models (Fig. 5) could be related to the disruption of the membrane in a clawing movement, as the C-terminal residues (MDiS) would move toward the plane of the iFace. Such a movement could pull phospholipids out of the membrane, disrupting its integrity, as hypothesized by Da Silva-Giotto *et al*.^25^. We calculated, along the direction of the tilt angle, a reduction in NM7 of active state PLA_2_-like proteins with fatty acids, tilt angles and MDiS approximations to the iFace (Fig. 5c and Supplementary Video S8). This approximation was evaluated by calculating the distance of the centroids of 121 Cβ and 125 Cβ to the plane of sulphates (dMDiSiFace). We calculated this distance using the plane equation with three sulphur atoms from the plane of sulphates in BnIV/myristic acid (shown in Fig. 2) after the dimers were superposed. The reduction of the tilt angles was correlated with dMDoSiFace and dMDiSiFace reductions (Fig. 5c). Therefore, the tilt variation among the crystal and NM-MD structures supports the hypothesis that reduced tilt angles correlate the MDoS and MDiS approximations to the toxin iFace.

### Action mechanism

**Figure 6:**
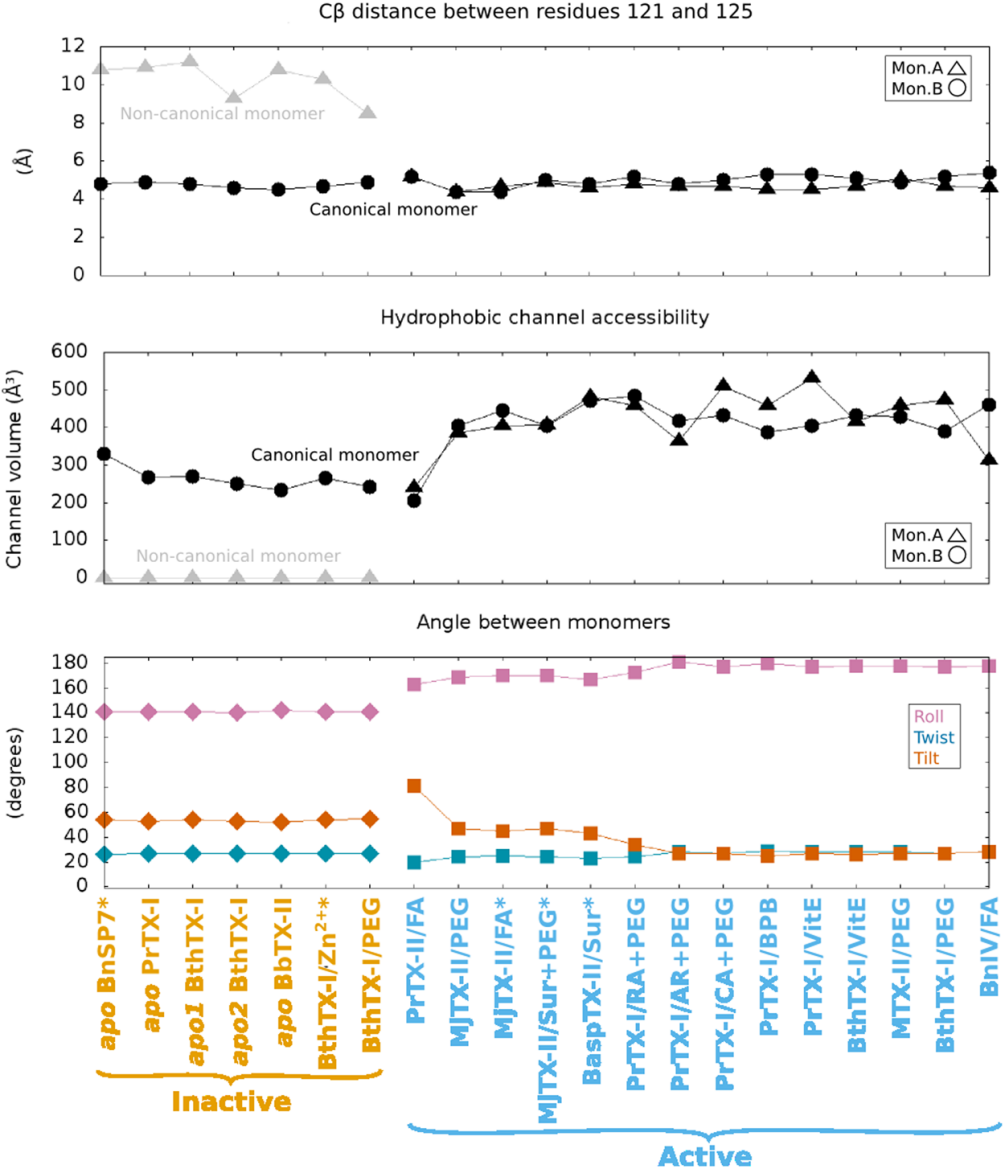
Distance between Cβs between residues 121 and 125, hydrophobic channel accessibility with the tunnel volume calculation, and monomer-monomer angles for all available bothropic PLA_2_-like toxins. The points refer to the structure shown in the abscissa of the third graph. In the first two graphs, monomers A and B are represented as ▴ and ⦁, respectively. In the monomer-monomer angle chart, the roll, twist, and tilt angles are coloured in magenta, vermilion and dark blue, respectively. The structures are coloured according to state, with inactive in orange and active in light blue. Toxins in an inactive state feature asymmetrical monomers, as one is non-canonical monomer with a MDiS distance greater than 8 Å and has hydrophobic channel closed (tunnel with volume 0) colored in grey in first two graphs, and its orientation relative to the other monomer is not symmetrical, as the numbers are far from 0 and 180°. Toxins in the active state possess both canonical monomers with MDiS distances close to 5 Å and both hydrophobic channels open, and the monomer-monomer orientation tends to a symmetrical relationship, as the angles tend to be either 0 or 180°. The abbreviations are related to polyethylene glycol (PEG), suramin (sur), α-tocopherol (VitE), and fatty acid (FA).

Based on these global and local analyses, we can better describe the various states of bothropic PLA_2_-like protein structures (Fig. 6). The inactive state of bothropic PLA_2_-like protein has monomers in an asymmetrical conformation. One monomer (A) is in a non-canonical conformation that does not have a MDiS cluster (dMDiS > 9 Å), and the hydrophobic channel is inaccessible (as observed by grey triangles of the orange structures in the dMDiS and the hydrophobic channel accessibility graphs shown in Fig. 6). The geometric relationship of the monomers is far from a symmetrical orientation, as the roll is distant to 180° (observed from angles of the orange structures in the monomer-monomer angle graph shown in Fig. 6). Both monomers of these structures lack hydrophobic molecules inside the hydrophobic channel.

Due to the entrance of a hydrophobic molecule in the accessible toxin channel, the monomers are reoriented, with an increased roll angle towards 180°, to a more symmetrical relationship, leading to toxin activation. Active state models feature both monomers as being symmetrical with MDiS (dMDiS < 6 Å), and both hydrophobic channels are accessible (observed on the light blue structures in the MDiS distance and hydrophobic channel accessibility graphs in Fig. 6) and occupied by hydrophobic molecules. Despite these similarities, they differ according to their tilt angles in three primary categories, which are interchangeable according to NM calculations. Our data support that these differences are not related to a specific protein but are exclusive to a minimum energy level reached in the crystal packing, and different PLA_2_-like toxins may achieve the conformations of one another in solution. The reduction of the tilt angles precludes the MDiS approximation to the iFace, supporting this clawing movement as a membrane disruption mechanism (Supplementary Video S8).

In conclusion, we observed new structural features of PLA_2_-like toxins by measuring the MDiS distance, hydrophobic channel accessibility and geometric orientation between monomers from the available crystal and NM-MD structures. Our proposed geometrical description, with Euler angles, has proven to be useful for generating a detailed description of the dimeric arrangement of different PLA_2_-like toxin models, as we identified an inactive state and three active states. We also used this general description to interpret interchain movements according to MD studies after validating the model pairing by RMSD calculations. Moreover, this methodology can be used as a primary tool to characterize geometrically homodimeric protein models by quickly separating structures into groups and using the angles to intuitively interpret global flexibility, describing the mechanism of action.

## Methods

### Structures

The following structures, with the Protein Data Bank identification codes in parentheses (PDB id), were used: *apo1* BthTX-I (3HZD), *apo2* BthTX-I (3I3H), BthTX-I/Zn^2+^ (4WTB)^24^, BthTX-I/PEG4k [polyethylene glycol with an average weight of 4000 g/mol) (3IQ3)]^13^, BthTX-I/PEG400 (2H8I)^21^, BthTX-I/α-tocopherol (3CXI), PrTX-I/α-tocopherol (3CYL), *apo* PrTX-I (2Q2J)^22^, PrTX-I/BPB (2OK9)^37^, PrTX-I/rosmarinic acid+PEG330 (3QNL)^38^, PrTX-I/caffeic acid+PEG4k (4YU7), PrTX-I/aristolochic acid+PEG4k (4YZ7)^39^, PrTX-II/*n*-tridecanoic acid (1QLL)^40^, *apo* BnSP-VII (1PA0)^26^, *apo* BbTX-II (4K09), MTX-II/PEG4k (4K06)^14^, BaspTX-II/suramin (1Y4L)^41^, BnIV/myristic acid (3MLM)^42^, MjTX-II/stearic acid (1XXS)^43^, MjTX-II/PEG4k (4KF3)^15^, and MjTX-II/suramin+PEG4k (4YV5)^44^. The structures BthTX-I/BPB (3I03), *apo* BthTX-I (3I3I), *apo* BnSP-VI (1PC9), *apo* BaspTX-II (1CLP), and BbTX-II (4DCF) were not included because in the first two, their asymmetric unit (ASU) content is composed of a monomer, and in the others, the resolution was lower than or equal to 2.5 Å.

### Bioinformatic tools

The *software CAVER 3*.*0* (*command line version*)^45^ was used to characterize the toxin hydrophobic channel of the PLA_2_-like crystallographic models. Hydrophobic channels are referred to as tunnels by this algorithm. To improve the convergence of the different tunnels, the probe and shell radii were increased to 1.8 and 4, respectively. The calculated tunnels were manually inspected in *PyMOL* (The PyMOL Molecular Graphics System, Version 1.7 Schrödinger, LLC.), and their volumes were calculated using the diameter and length of the tunnel. When PEGs or fatty acids were present in the model, the tunnel whose path filled this molecule was chosen. *PyMOL* and VMD (http://www.ks.uiuc.edu/Research/vmd/)^46^ was used to create cartoon and stick images.

### Geometrical description between identical monomers

The geometric orientation between the monomers was measured by superposing one on the other and converting the matrix rotation into Euler angles (following the sequence 3,2,1 using the formulas from Section 8.11 of Diebel’s publication^47^). This is the most intuitive 3D rotation description as it uses each of the 3 axes once, such as the Tait-Bryan angles (row, pitch and yaw) used in aeronautics^48^.

Prior to the superposition calculations, the models were placed into immutable and standard orthogonal axes that relate structure to function. As the PLA_2_-like protein iFace forms a plane that is mostly composed of the N- and C-terminal regions of the protein, which are perpendicular to the two antiparallel α-helices (H2 and H3) (Fig. 2a), these two helices were used as references for two of the axes. To obtain the first axis in the direction and centre of the largest α-helix, the centroid of 7 consecutive carbon α (named herein as Cc7 and shown as grey spheres in Fig. 2b) was calculated. Approximately seven residues complete two α-helix turns. To facilitate the placement of different models in a common set of orthogonal axes, three Cc7s with small fluctuations were selected from the two longest helices, Cc7/48, Cc7/98 and Cc7/104, which had RMSF of 0.13, 0.08, and 0.08 Å, respectively (grey line in Fig. 1). The RMSF of Cα (black line in Fig. 1) was calculated after monomers were separated and superposed to a template (monomer A of BthTX-I/PEG4k). The RMSF of Cc7 was obtained using previous superposed structures. Cc7/98 was chosen as the origin, and Cc7/98 to Cc7/104 was used as the X-axis (magenta axis in Fig. 2b). The Y-axis was constructed as the perpendicular vector to the X-axis inside the plane composed of the X-axis and coordinate Cc7/48 (blue axis in Fig. 2b). One of the monomers was chosen as a reference to calculate the rotation matrix that was used to superpose it onto the other monomer.

The *software lsqkab* ^49^ was used to generate the required distances to calculate the RMSF and obtain the matrix rotation of the superpositions. The Ti, Ts and R angles were obtained from the rotation matrix. For the translation calculation, the distance between the centre of gravity of each monomer was obtained using the function gravity of mass available in *PyMOL*.

### Normal mode analysis

The LF NMs movements of the bothropic PLA_2_-like toxin containing fatty acids in the hydrophobic channel were calculated by the CHARMM v.36b1 program^50^ and CHARMM36 force field^51^. The inactive state model inact and active state models acti1–3 were selected. As some of these models have PEGs inside the toxin hydrophobic channels, the ligands in acti1 and inact canonical monomers were replaced by myristic acids from the BnIV/myristic acid model after monomer-monomer Cα superposition. Since inact only has one canonical monomer, only one fatty acid was introduced in the structure. Inact and acti1 models absent of fatty acids were included in NM calculations. The topology and parameter files were generated by the CHARMM-GUI server (www.charmm-gui.org)^52^ by employing an additional energy minimization. The conjugated gradient algorithm was applied with harmonic constraints that were progressively decreased from 250 to 5 kcal/mol·Å^2^, with 100 steps of minimization at each decrease. The constraints were removed to carry out an additional 10,000 conjugated gradient steps, followed by 300,000 steps of the basis Newton-Raphson algorithm. The final minimized structure was used to calculate the three lowest frequencies NMs (7–9) using the VIBRAN module of CHARMM for the structures.

The structures were generated along each NM based on a short MD simulation at a low temperature (30 K) using the VMOD facility of CHARMM followed by a minimization afterwards. A maximum displacement range of 6.0 Å was established for each direction of the NM with a 0.1 Å projection step based on the values of the mass-weighted root mean square. For each of these steps, a constant harmonic force was applied over the Cα atoms (increasing from 1,000 until 10,000 kcal/mol·Å^2^), and a short MD simulation (1 ps) was carried out after each constant value, totalling 10 ps of simulation. The final structures were obtained by an additional 1,000 steps of conjugated gradient energy minimization maintaining the restraints. The miscellaneous mean field potential facility of CHARMM of the final structures was used for energy validation.

To relate NM to function, the distance of the essential cluster of residues to the PLA_2_-like iFace was calculated. This was accomplished by extracting the plane equation from the coordinates of 3 sulphurs of sulphates interacting with the protein iFace and calculating the distance from the chosen residues. To compare different NM motions in respect to the different states of PLA_2_-like proteins crystallographic structures, RMSD was calculated using these and the structures from NM analysis. The RMSD was calculated with *lsqkab* program. To illustrate NM motions, the principal component was calculated using the quasi-routine of the module VIBRAN of CHARMM to obtain the covariance matrix o Cα atomic displacements (according to Floquet *et al*.^53^).

## Electronic supplementary material

Supplementary Information

Supplementary Video S5 - Video 1

Supplementary Video - S6 - Video 2

Supplementary Video S7A - Video 3

Supplementary Video S7B - Video 4

Supplementary Video S7C - Video 5

Supplementary Video S8 - Video 6
