# Supplementary material for: PLA2-like proteins myotoxic mechanism: a dynamic model description
Source: Sci Rep. 2017 Nov 14;7:15514. doi: 10.1038/s41598-017-15614-z (PMC5686144; doi:10.1038/s41598-017-15614-z)
Supplement: Supplementary file 1 — Supplementary Information [file 41598_2017_15614_MOESM1_ESM.doc]

PLA_2_-like proteins myotoxic mechanism: a dynamic model description

Rafael J. Borges; Ney Lemke; Marcos R. M. Fontes

**SUPPLEMENTARY MATERIAL**


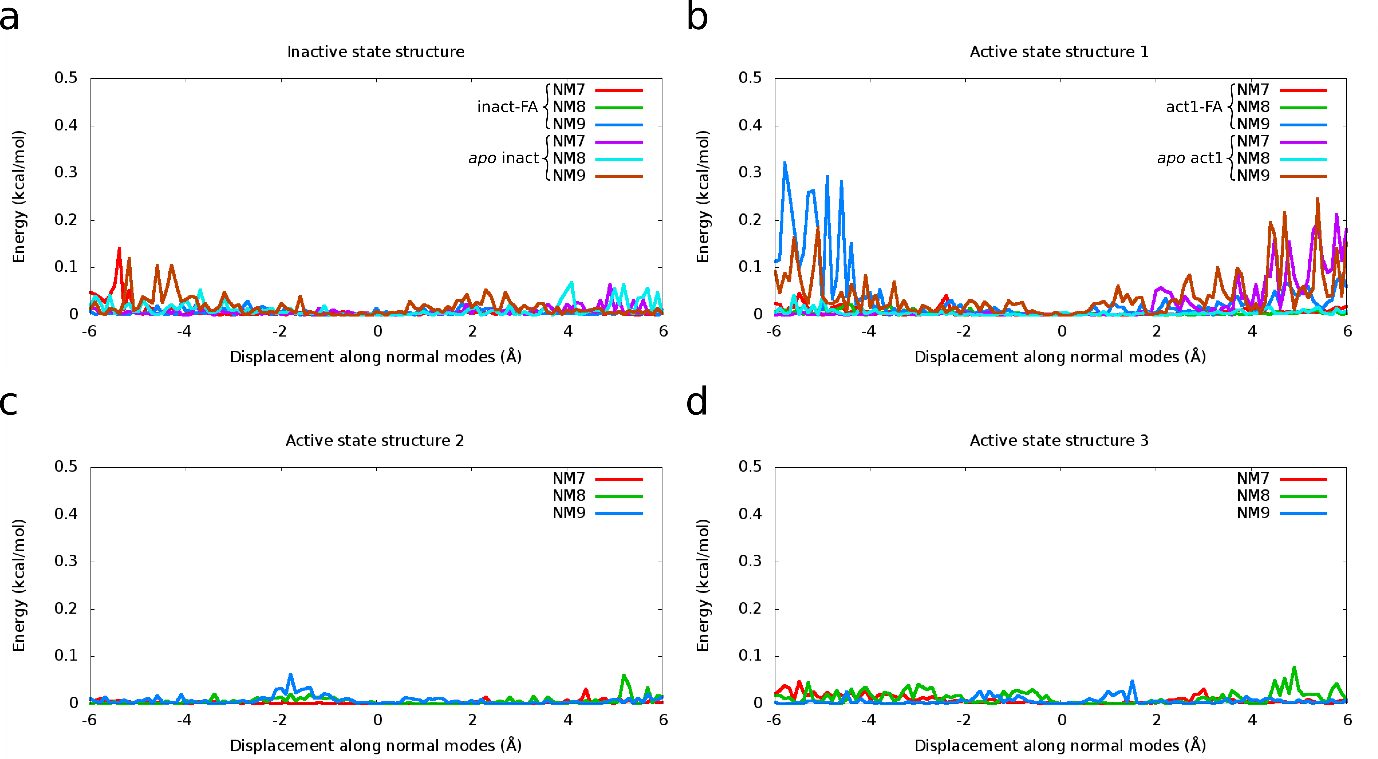


**Supplementary Fig.S2 -** Δpotential energy (kcal/mol) of minimized displaced structures along normal modes 7–9. Inactive (**a**), active 1 (**b**), 2 (**c**), and 3 (**d**) state structures are BthTX-I/PEG400 (PDB id: 2H8I), BthTX-I/PEG4k (PDB id: 3IQ3), MjTX-II/stearic acid (PDB id: 1XXS), and PrTX-II/*n*-tridecanoic acid (PDB id: 1QLL), respectively.


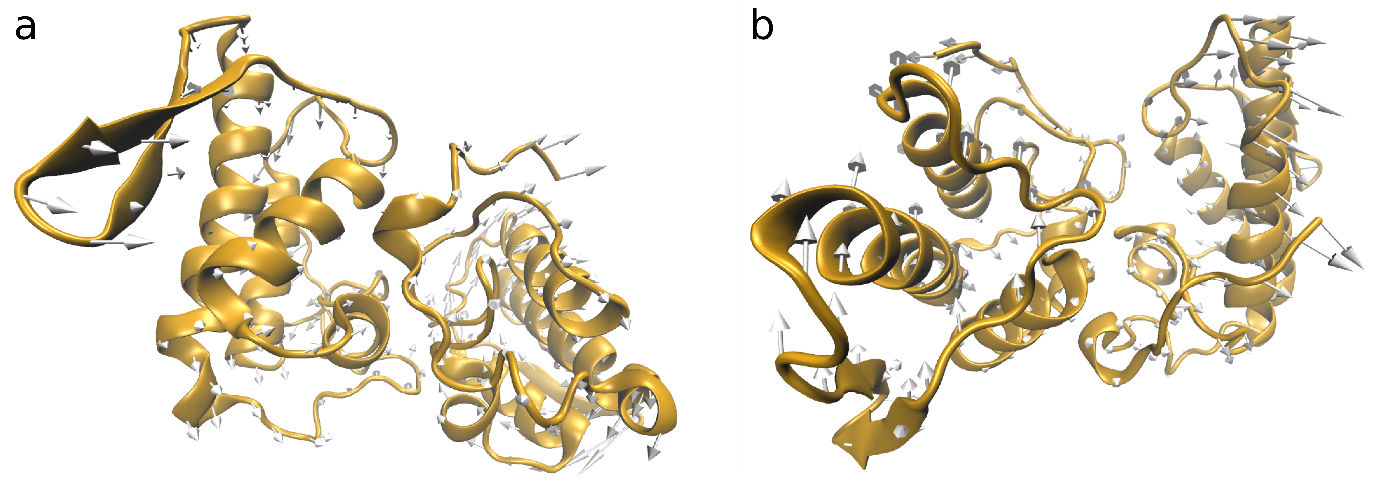


**Supplementary Fig. S4** - Activation motion of **inactive state** structure (orange cartoon) described by -3 Å displacement of normal mode 9, which is represented by arrows. **A** and **b** show the same representation with a 90^o^ rotation in the horizontal axis.

**Supplementary Video S5 -** Activation motion of the **inactive state** structure of PLA_2_-like proteins represented by orange cartoon. Motion is described by minimized structures along 0 to -3.1 Å displacement of normal mode 9 of BthTX-I/PEG400 (PDB id: 2H8I). Crystal structures of the BthTX-I/PEG400 and **active state** (BthTX-I/PEG4k – PDB id: 3IQ3) are represented by transparent orange and blue cartoon, respectively.

**Supplementary Video S6 -** Inactivation motion of the **active state** structure of PLA_2_-like proteins represented by blue cartoon. Motion is described by minimized structures along 0 to -2.7 Å displacement of normal mode 8 of BthTX-I/PEG4k (PDB id: 3IQ3). Crystal structures of the BthTX-I/PEG4k and **inactive state** (BthTX-I/PEG400 – PDB id: 2H8I) are represented by transparent blue and orange cartoon, respectively.

**Supplementary Video S7 -** Motion of conversion between **active state** structures (blue cartoon). In **a**,motion of acti1 (BthTX-I/PEG4k - PDB id: 3IQ3) is represented by minimized structures along 0 to -5.4 Å of normal mode 7displacement. In **b**, motion of acti2 (MjTX-II/fatty acid – PDB id: 1XXS) is represented by minimized structures along 0 to -3.8 Å of normal mode 7displacement. In **c**, motion of acti3 (PrTX-II/fatty acid – PDB id: 1QLL) is represented by minimized structures along 0 to -4.5 Å of normal mode 7displacement. Crystal structures of **active state** of BthTX-I, MjTX-II and PrTX-II are represented by transparent cartoon colored vermillion, orange, and yellow, respectively.

**Supplementary Video S8 -** Claw motion ofthemyotoxic mechanismof PLA_2_-like proteins. Membrane disruption site approximates and penetrates the membrane plane colored in dark orange. This motionis described by structures of BthTX-I (PDB id: 3IQ3) minimized along 0 to 6.0 Å normal mode 7displacement. PLA_2_-like protein is represented by a light blue cartoon, MDiS, by yellow sticks, and head of phospholipids by orange spheres.

| PDBs | Ligands | Hydrophobic Channel | | iFace | | MDiS | | Nt|H1|lp (outer) | | R34 | | Bsh | | H2 | | H3 | | Cterm | | Crystal System |
| --- | --- | --- | --- | --- | --- | --- | --- | --- | --- | --- | --- | --- | --- | --- | --- | --- | --- | --- | --- | --- |
| ASYMMETRICAL MONOMERS COMPLEXED TO NATURAL MOLECULES | | | | | | | | | | | | | | | | | | | |  |
| **2Q2J** | **1 sulfate / 2 TRIS** |  |  | AB | |  |  |  |  |  |  |  |  |  |  |  |  | A | | Trig |
| **2H8I** | **1 PEG** | A | |  |  |  |  |  |  |  |  |  |  |  | |  |  |  |  |
| SYMMETRICAL MONOMERS COMPLEXED TO FATTY ACIDS OR SIMILAR MOLECULES | | | | | | | | | | | | | | | | | | | |  |
| **4KF3** | **4 PEGs/ 6 isopropanol** | AB | | AB | |  | | A | | AB | | A | |  | |  |  |  |  | Ort |
| **1XXS*** | **4 stearic acids/ 5 sulfate** | AB | | AB | |  | | A | | AB | | A | |  | |  | | A | |
| **3CYL** | **2 α-tocopherols / 1 PEG/ 5 sulfates** | AB | | AB | |  | | B | | A | | B | |  | | A | |  |  | Mon |
| **3CXI** | **2 α-tocopherols / 1 PEG/ 4 sulfates** | AB | | AB | |  | | B | |  |  | B | |  |  | A | |  |  |
| **4K06** | **3 PEGs / 5 sulfates** | AB | | AB | |  | | B | | A | | B | |  | | A | |  |  |
| **3IQ3** | **3 PEGs / 2 sulfates** | AB | |  |  |  |  | B | | A | | B | |  | | A | |  |  |
| **3MLM** | **2 myristic acids/ 4 sulfates** | AB | | AB | |  | |  | | AB | |  |  |  | |  |  |  |  |
| **1QLL** | **2 N-tridecanoic acids** | AB | |  |  |  | |  | |  | |  | |  | |  | |  |  |
| COMPLEXES WITH INHIBITORS | | | | | | | | | | | | | | | | | | | |  |
| **4WTB*** | **3 zinc / 2 sulfates** | A | B | AB | | AB | |  |  | A | |  |  | A | |  |  |  | | Trig |
| **4YV5*** | **2 suramin / 3 PEGs / 7 sulfates** | AB | | AB | AB | AB | | AB | A | AB | | AB | A |  | |  |  | AB | A |  |
| **1Y4L*** | **1 suramin / 2 PEGs/ 5 isopropanol** | AB | AB | AB | |  | | AB | | AB | |  |  | AB | B |  | |  | |
| **3QNL** | **1 rosmarinic acid / 1 PEG/ 8 isopropanol** | B | AB | A | | B | | A | |  |  | B | | B | | A | |  |  |
| **4YZ7** | **1 aristolochic acid / 1 PEG/ 5 sulfates** | B | | B | | A | | B | | AB | |  |  |  |  | A | |  |  |
| **4YU7** | **4 caffeic acids/ 3 PEGs/ 1 sulfate** | AB | | AB | |  |  | A | | A | | A | |  | | A | |  |  | Mon |
| **2OK9** | **2 BPBs/ 2 isopropanol** | AB | AB |  |  |  |  |  | |  |  |  |  |  | |  |  |  |  |

Supplementary Table S1 - Summary of binding regions of ligands with available bothropic PLA_2_-like crystallographic models

In PDBs column, font colored in orange, red and light blue are presentation of asymmetric, exception and symmetric PLA_2_-like properties, respectively. In the other column, negative molecules, PLA_2_-like protein inhibitors, and fatty acids (and similar) are colored in orange, green, and light blue, respectively. * PDBs had their chain letter A and B inverted. The abbreviations refers to protein region that interacts to the membrane (iFace), Membrane disruption site (MDiS), exposed atoms of residues of N-terminal α-helix and loop (Nt| H1|lp (outer)), arginine at position 34 (R34), β-sheets (Bsh), helix 2 (H2), helix 3 (H3), C-terminal (Cterm), Trigonal (Trig), Orthorhombic (Ort), and Monoclinic (Mon).For the site calculation, we considered the basic residues 16-17, 20, 115 and 118for iFace; 120-126 for MDiS; 1-15, 18-19, 21-24 for Nt| H1|lp (outer); 72-88 for Bsh; 35-47,49-69 for H2; 91-109 for H3; and 110-114, 116-117, 119 and 127-133 for Cterm. The hydrophobic channel herein is described as the proposed tunnel, which is composedof the inner part of N-terminal residues and the calcium binding loops.

| Structure | Normal Mode | Minimum RMSD [Correspondent to  structure along displaced NM (Å)] | | | |
| --- | --- | --- | --- | --- | --- |
|  |  | 2H8I | 3IQ3 | 1XXS | 1QLL |
| *Apo* Inactive  *Apo* BthTX-I/FA | 7 | 1.7 | 3.6 | N.D. | N.D. |
|  | 8 | 1.1 | 3.0 | N.D. | N.D. |
|  | **9** | **1.3** | **1.9 (-3.1)** | N.D. | N.D. |
|  |  |  |  |  |  |
| Inactive-FA  BthTX-I/FA | 7 | 1.7 | **3.8** | N.D. | N.D. |
|  | 8 | 1.2 | **2.8** | N.D. | N.D. |
|  | 9 | 1.3 | **3.0** | N.D. | N.D. |
|  |  |  |  |  |  |
| Active3  *Apo* BthTX-I | 7 | 1.9 | 1.5 | N.D. | N.D. |
|  | **8** | **1.7** | **1.8** | N.D. | N.D. |
|  | 9 | 2.7 | 1.6 | N.D. | N.D. |
|  |  |  |  |  |  |
| Active3  BthTX-I/FA | **7** | 2.3 | 1.4 | **1.6 (0.6)** | **2.0 (5.4)** |
|  | **8** | **1.5 (-2.7)** | **1.3** | 2.1 | 5.4 |
|  | 9 | 2.8 | 1.3 | 1.9 | 5. 7 |
|  |  |  |  |  |  |
| Active2  MjTX-II/FA | **7** | N.D. | **2.0 (3.0)** | **1.5** | **2.0 (-3.8)** |
|  | 8 | N.D. | 2.6 | 1.3 | 4.3 |
|  | 9 | N.D. | 2.6 | 1.4 | 3.6 |
|  |  |  |  |  |  |
| Active1  PrTX-II/FA | **7** | N.D. | **3.3 (-4.5)** | **3.1 (-4.5)** | **1.0** |
|  | 8 | N.D. | 6.3 | 4.9 | 1.1 |
|  | 9 | N.D. | 5.5 | 4.1 | 1.0 |

**Supplementary Table S3** - Summary of superposition of the representatives of **inactive** and **active state** models to generated models by NM-MD

Number in parenthesis refers to which structure along given Normal Mode refers to minimum. Inactive, active1, active2, and active3 refers to calculation from structures BthTX-I/PEG400 (PDB id: 2H8I), BthTX-I/PEG4k (3IQ3), MjTX-II/stearic acid (1XXS), PrTX-II/n-tridecanoic acid (1QLL), respectively. Abbreviation FA refers to fatty acid, and N.D. to not determined. Bold number refer to analysis used in this article.
